# Dr. Griffith’s Address

**Published:** 1851-10

**Authors:** 


### DENTAL REGISTER OF THE WEST.

Vol. V. OCTOBER, 1851. No. 1.

### Original Communications.

Surgeon Gen'l's Office LIBRARY 22294 Washington, D.C.

### DR. GRIFFITH'S ADDRESS

*Before the Seventh Annual Meeting of the Mississippi Valley Association of Dental Surgeons, held in Louisville, September 11th, 12th and 13th, 1851.*

I WELCOME you, gentlemen, to Louisville. It is a source of much pleasure to me to see so many members of the Association present at this its second meeting in this city. It speaks much for the past sittings of the society, and it argues well for its prospective usefulness, that a number so respectable, both in character and talents, could be convened at this season of the year. I trust that the spirit which led to the formation of the society, and which has prevailed at its sittings hitherto, will be manifest here, and will continue to grow and to expand until the profession in the whole Valley shall feel its influence. It seems to me that we have great reason to congratulate ourselves on the present condition of Dentistry. All of you are acquainted with the history of our science, and can contrast the rude instruments and contrivances of the past, offsprings, as they were, of a rude knowledge, with the elegance and abundance of the present, the offspring and the concomitants of a better cultivation and a higher knowledge. This past, too, is not a very distant past; there are those here, still active, who have watched the progress of this improvement, and who have helped to bring it about. It is a little remarkable that while that branch of medicine and surgery, called Dentistry, must have been among the first practiced in the family of man, it should be about the last to be cultivated to a degree commensurate with its importance. It was left to barbers, tinkers and coblers, as was indeed much also of surgery; its practice was considered disreputable, and its practitioners were not admitted to the fellowship of those even who devoted themselves to the kindred branches of science. It seems strange that diseases which have so much to do with the comfort and the well-being of individuals of every age and condition, and (what to a certain portion is quite as important,) with their beauty or deformity, should have been so long neglected. But all this is changed; the time has gone by when fellows, too lazy to work, could turn Dentist, and with their forceps and file and gold foil set about their murderous business of pulling, rasping and filling. Society will no longer permit this. Men of character and of extensive attainments have entered the field and driven out these miserable pretenders. Society expects more from our profession—a thorough education, a knowledge of the laws of health and disease, and great mechanical skill; and it is we who have taught society this. The public mind has not led the way in this improvement, but it is we who have educated it to know the difference between good dentistry and bad, or rather between good dentistry and no dentistry.

Dental science is unquestionably very largely indebted for its improved state to thc labors of physicians and surgeons; of those especially who have cultivated physiology and surgical pathology. Some of the most distinguished men in the medical and surgical professions have devoted themselves to the investigation of the anatomy and physiology of the teeth, and the diseases of the mouth and the organs of mastication have received their full share of benefit from the progress of medical and surgical science.

While we cheerfully acknowledge this indebtedness, it must not be supposed that our profession has not repaid in kind; that it has not reflected back some of the benefits which it thus derived. All departments of the healing art are so bound together, that each aids in the advancement and improvement of the others. But in the case of dentistry it has conferred one single boon on medicine and surgery that will more than counterbalance all the benefits hitherto derived from them. The successful employment of anæsthetic agents is wholly due to our profession.

It was to relieve the pain attendant on many dental operations that thei rinfluence was first sought; it is to a member of our own profession, to a practitioner of dentistry, that science and humanity are indebted for their successful introduction. To you, who can appreciate it so well, I need not speak of the vast importance of Dr. Morton's discovery. Its value is incalculable. Let the thanks and the blessings, swelling up from suffering humanity everywhere and through all coming time, speak its value and be its eulogy.

It is no part of my purpose to recapitulate the achievements of our professional brethren in overcoming disease or removing deformity, it would be useless, as they are known as well to you as myself. There is still another reason, while these achievements are justly and naturally a source of pride and gratification to us, we should be careful not to fix our attention on them to the exclusion of what is apparently of less moment. It is a constant subject of complaint in medicine and surgery, that in the search for something unusual, in the desire of a brilliant operation, the small every day things, those which have to be daily and hourly combated, and which constitute by far the larger portion of the practitioner's duty, are too often wholly overlooked. The same is true, I think, of dentistry. There is too great a tendency to neglect the common and familiar for that which is novel and striking. I would not wish to repress the desire for distinction, which is both natural and proper, and has lead and will hereafter lead to important results. It is only when these desires degenerates into mere eagerness to achieve a position or reputation by some bold or novel proceeding, that I would have it attempered and restrained. In the search for novelties, the small beginnings of disease are lost sight of; while Rush's great maxim "to oppose the beginnings of disease," is as applicable to dentistry as to medicine and surgery proper.

To illustrate my meaning better I will call attention to some points of practice, the importance of which, according to my observation, is not always fully appreciated by us, and of course is not by the public. For the public, as I have already observed, take their cue from us, and what we regard as trivial they will regard as still more trivial. One is the very common operation of removing what is called *tartar* from about the teeth. It is too often carelessly done; the larger masses, the more obvious or more salient accumulations are removed; but it is not pursued closely enough, is not hunted out and dislodged from all its hiding places. I have seen recently a marked instance of bad effects from this neglect, at least a state of things that could be traced to no other cause, and with your permission I will relate to you the case:

Mr. Wm. W., a gentleman, living four miles from Louisville, called on me the 1st of April, 1851, to consult me in reference to his teeth, and the propriety of extracting some roots, which he supposed to be the cause of his great suffering. He was about fifty-five years old, and of a strong constitution. I inquired of him if he could assign any reason for the ulceration of his gums; if he had at any time been diseased from any cause; he said he could not recollect that he had. I then asked him if he had been using strong acids previous to the decaying of his teeth, which he answered in the negative. I then asked him to explain in what way his teeth commenced going. He remarked, the first thing I noticed was my teeth commenced getting very dark near the gums, and the gums getting sore, and two or three years afterward my teeth commenced crumbling and breaking off; and, about six months ago, my jaws became very painful, my gums swelled and have been discharging matter ever since, and I thought I would die if I could get no relief, and I was advised to call and get your advice. I examined his mouth carefully; gums badly ulcerated and mattering freely. I extracted the two incisores and cuspidates and bicuspids of the right side above, all of which was very much diseased. Supposing that this would give relief, I discharged him, directing him to use an astringent wash, of sulphate of zinc and myrrh, and if he continued to suffer to call on me in the course of a week or ten days. Three weeks from that time he called again, and appeared to be suffering more, if possible, than before. Upon examining his mouth I found little or no change in appearance, and when I touched the molar teeth they gave him much pain, and appeared to be entirely detached from the bone. I then introduced a silver probe and discovered the alveolar processes all exfoliated. I extracted the two molars, then made a free incision with my lancet; and, with a pair of dressing forceps, I removed three pieces of bone, the first piece was one and an eighth of an inch in length, the second three fourths of an inch and the third one half an inch. I then directed him to get a small syringe and inject his gums freely with the wash I had given him, and call on me again in two weeks. He did not call until the 9th of August, and his reason for not calling was that he supposed his mouth was well; but, upon examination, I found a piece of the bone projecting slightly through the gums; which I removed, and with this exception his mouth was entirely well. All this I attributed, in the first place, to a neglect of cleaning his teeth, and in the second place in not extracting the roots of teeth in time.

Another matter, which is often regarded with too little attention, is the extraction of the diseased teeth and their roots, and all such teeth as cannot be restored to a moderate degree of health. It is of great importance, I know, to save a tooth, and a patient is grateful for such service; still, in the desire to achieve this, we ought not to risk reputation and the welfare of the patients as I know that I myself have done. The immediate extraction of all teeth which cannot be restored to health, and of all remains of decayed teeth, ought to be insisted on as saving future trouble, both to ourselves and patient. They are constant sources of irritation, and on exposure of the individual to the ordinary causes of disease, we may and do have inflammation set up, leading to extensive suppuration, to abscess, to ulceration and even to necrosis or caries of the alveolar process, or of the body of the maxillary bone and accompanied with an immense amount of constitutional irritation, as in the case I have just related, which, by timely attention, might have saved the individual a vast amount of suffering and trouble. I need not multiply these instances, as many others will suggest themselves to you. I desire only to impress on the minds of the younger practitioners especially, the importance of paying close attention to what may seem to them even trivial. He is the best dentist, the most useful, and best fulfills his obligations to the public, who is careful of minor matters.

Finally, gentlemen, since dentistry has taken its proper place among the sciences of this enlightened age, and thrown from it most of the prejudices with which ignorance had so long kept it trammeled, let us realize the responsibility of guarding it against the malignant influences which may spring from the practice of unskillful practitioners and be brought to bear against the science. I feel gratified to know that our profession can boast of its scientific men, and happy am I to have the counsel and influence of such talent.

